# Optimal Control of an Electromechanical Energy Harvester

**DOI:** 10.3390/e27030268

**Published:** 2025-03-05

**Authors:** Dario Lucente, Alessandro Manacorda, Andrea Plati, Alessandro Sarracino, Marco Baldovin

**Affiliations:** 1 Department of Mathematics & Physics, University of Campania “Luigi Vanvitelli”, Viale Lincoln 5, 81100 Caserta, Italy; dario.lucente@unicampania.it; 2 CNR Institute for Complex Systems, Università Sapienza, P.le A. Moro 5, 00185 Rome, Italy; marco.baldovin@cnr.it; 3 Laboratoire de Physique des Solides, Université Paris-Saclay CNRS, 1 Rue Nicolas Appert, 91405 Orsay, France; andrea.plati@universite-paris-saclay.fr; 4 Department of Engineering, University of Campania “Luigi Vanvitelli”, Via Roma 29, 81031 Aversa, Italy; alessandro.sarracino@unicampania.it

**Keywords:** optimal control, energy harvesting, stochastic processes

## Abstract

Many techniques originally developed in the context of deterministic control theory have recently been applied to the quest for optimal protocols in stochastic processes. Given a system subject to environmental fluctuations, one may ask what is the best way to change its controllable parameters in time in order to maximize, on average, a certain reward function, while steering the system between two pre-assigned states. In this work, we study the problem of optimal control for a wide class of stochastic systems, inspired by a model of an energy harvester. The stochastic noise in this system is due to the mechanical vibrations, while the reward function is the average power extracted from them. We consider the case in which the electrical resistance of the harvester can be changed in time, and we exploit the tools of control theory to work out optimal solutions in a perturbative regime, close to the stationary state. Our results show that it is possible to design protocols that perform better than any possible solution with constant resistance.

## 1. Introduction

Control theory deals with systems whose evolution can be externally steered by varying the value of some parameters. By suitably choosing time-dependent protocols for them, it is possible to drive the system between pre-assigned end states: a typical goal is to do so while minimizing a certain cost function (or maximizing some sort of reward). The theory was initially developed for deterministic systems [1,2,3], and later extended to quantum dynamics involving probabilistic descriptions: in this case, even reaching the pre-assigned final state, which is given in terms of a probability distribution, is a non-trivial problem (usually referred to as *shortcut to adiabaticity*) [4]. During the last two decades, these ideas have found wide application in the context of stochastic systems [5,6]. The paradigmatic case of colloidal particles trapped in confining potentials has been studied both in the overdamped [7,8,9] and underdamped regime [10,11,12,13], as well as in the presence of non-harmonic confinements [14,15,16]. Recent studies have extended the focus to out-of-equilibrium systems, including the so-called Brownian Gyrator [17], stochastic dynamics with non-Markovian noise [18], stochastic resetting [19,20], active particles [21,22,23,24], granular materials [25,26] and friction [27]. All these results strongly suggest that the framework of control theory is versatile enough to address complex real-life applications, such as those related to the optimization of energy harvesting in the presence of random fluctuations.

Energy harvesters are devices that can collect available energy from environmental sources, exploiting a huge gamma of different physical mechanisms [28]. The optimization of energy harvesting strategies represents a central issue for the maximization of the extracted power. From the theoretical perspective, for the mathematical description of these systems a central role is played by stochastic models, which mimic the random nature of fluctuations in the often unpredictable and uncontrolled environmental conditions.

In this work, we consider a linear stochastic model based on an underdamped Langevin equation that has been shown to describe with very good accuracy two kinds of energy harvesters, based on piezoelectric [29,30,31,32] and electromagnetic [33] effects. Piezoelectric materials [34] can convert mechanical stress into electrical energy, and therefore they are used to design devices to harvest energy from environment vibrations. For instance, the small currents produced can be exploited to feed sensors in wireless sensor networks. Electromechanical energy harvesters [35] are typically realized with a permanent magnet, connected to the housing by a spring, and subjected to vibrations. The relative displacement with respect to a coil fixed to the housing and connected to an electrical load produces the conversion of the mechanical energy into electrical energy. Electromechanical harvesters are the electrical counterpart of ratchets, i.e., devices able to convert environmental fluctuations into directed motion [36,37,38,39]; the optimization problem equally applies for ratchets [40,41,42], and the strategy introduced in this work is a suitable candidate for this challenge.

At variance with previous works [29,30,33], where optimization at fixed parameters was studied, we assume here that the resistance appearing in the load circuit of the energy harvester can be varied in time (i.e., we imagine substituting it with a controllable potentiometer). We derive quite general equations for the optimal control of a class of stochastic processes that includes the considered model as a particular case. We then specialize to the case of small changes in the control parameter: this choice allows us to solve these equations perturbatively. Remarkably, we are able to identify new control protocols that perform better than the previously known optimal strategy, obtained by suitably fixing a constant value of the resistance. Our present results are obtained in a particular case, but they serve as a proof of principle for future, more exhaustive investigations.

The structure of this paper is as follows. In Section 2, we discuss the stochastic model describing the energy harvester that we want to optimize. In Section 3, we introduce the concepts of control theory that we need in order to maximixe the performance, and we carry out explicit calculations for a wide class of stochastic systems, including the one introduced before. A perturbative method for the solution of the problem is outlined. Section 4 is devoted to the discussion of the results obtained for some particular choices of the parameters. Finally, in Section 5 we draw our conclusions.

## 2. Energy Harvester Dynamics

We consider a theoretical model that has been shown to very well describe the behavior of electromagnetic and piezoelectric energy harvesters driven by broadband vibrations, not only for the average values of the main quantities of interest, but even at the fine level of their fluctuations [30,33]. In the simplest realization, both these systems consist of a movable part (permanent magnet or piece of piezoelectric material, respectively) that is subjected to vibration and is electromechanically coupled with the current flowing in the output electric circuit. We will focus here on the electromagnetic harvester, but our study can be easily extended to the piezoelectric case. In particular, the main variables appearing in the theoretical model are the magnet position *x* with respect to the coil, its velocity *v* and the current *I*. The forces acting on the magnet are due to a harmonic potential (representing the spring), the viscous friction of the air, the coupling with the current (Lorentz force, due to the interaction between the current *I* flowing into the coil and the induction field of the magnet) and the stochastic driving, which we describe as white noise. Therefore, in the linear approximation, the system is described by an underdamped Langevin equation for the magnet position, coupled with a deterministic equation that describes the time evolution of the current:

(1) $$\begin{array}{cc} \dot{x} & =v\\ M\dot{v} & =-k_{s}x-\gamma v-\theta I+M\xi \\ L_{C}\dot{I} & =\theta v-(R_{C}+R)I\\ \langle \xi \left(t\right)\xi \left(t^{\prime }\right)\rangle & =2D_{0}\delta \left(t-t^{\prime }\right). \end{array}$$

In the above equations, *M* represents the mass of the magnet, γ represents the viscous damping due to the air friction, $k_{s}$ is the elastic constant of the spring system, *I* is the current at the electrical terminals, $L_{C}$ and $R_{C}$ are the coil inductance and resistance, respectively, *R* is the load resistance of the output circuit and θ is the effective electromechanical coupling factor, taking into account the coil geometrical properties, number of turns and magnetic field strength. The coefficient $D_{0}$ quantifies the noise amplitude. We observe that in real systems this is not thermal noise but has a macroscopic origin (for instance, car or train vibrations). Moreover, we have neglected the Johnson–Nyquist thermal noise in the equation for the current because it is typically very small in energy harvesters. Finally, we note that the coupling between magnet velocity and electrical current can be interpreted as a feedback mechanism, and the model can be mapped into a single generalized Langevin equation with memory effect [30]. Other kinds of energy harvesters for which optimal control protocols have been studied exploit bi- or tri-stable potentials and are driven by white or colored noise [43,44,45,46,47,48].

The extracted power is obtained from the average of the square of the current flowing in the load resistance. Here, we consider the case of a time-dependent value of R=R(t),

(2) $$P_{harv}\left[R\left(t\right)\right]=\int_{t_{0}}^{tf}dt\;R\left(t\right)\langle I^{2}\left(t\right)\rangle .$$

Our goal is to find the optimal protocol R(t) that maximizes the extracted power.

## 3. Global Optimization

A global optimal control of the problem introduced above can be found through Pontryagin’s Maximum Principle (PMP) [2,3,49]. We will see that this strategy leads to a quite involved system of ordinary differential equations, which can be studied perturbatively by taking the stationary optimal solution as a reference point and expanding the dynamics around the corresponding stationary state.

### 3.1. Pontryagin’s Maximum Principle

Let us consider a dynamical system characterized by its *state* $x\in R^{d}$, following the evolution equation

(3) $$\dot{x}\left(t\right)=F\left(x\left(t\right),u\left(t\right)\right)\;,\;x\left(t_{0}\right)=x_{0}\;,\;x\left(t_{f}\right)=x_{f}\;,$$

with u∈U⊂R being the *control* applied to the system. The initial and final conditions, $x_{0}$ and $x_{f}$, respectively, are fixed, as well as the final time $t_{f}$. These constraints identify a fixed endtime and fixed endpoint problem, and the control u(t) must be able to satisfy them. One then aims at maximizing a *reward* functional *J* defined as

(4) $$J\left[u\right]=-\int_{t_{0}}^{t_{f}}dt\;L\left(x\left(t\right),u\left(t\right)\right)\;.$$

The reward is a functional of the applied control u(t); the *Lagrangian* term L(x,u) can depend on the control explicitly and implicitly through x(t).

PMP provides the necessary conditions for the global optimality of the control u(t): given the dynamics and the reward defined above, one can typically define a Hamiltonian function H(x,λ,u) as

(5) H(x,λ,u)=λ·F(x,u)−L(x,u),

with $\lambda \in R^{d}$ being called the *costate* variable of the problem. PMP states that if $u^{*}\left(t\right)$ is a globally optimal control, the following equations hold:

(6) $$\begin{array}{ccc} {\dot{x}}^{*}\left(t\right) & =+\partial_{\lambda }H\left(x^{*}\left(t\right),\lambda^{*}\left(t\right),u^{*}\left(t\right)\right)=F\left(x^{*}\left(t\right),u^{*}\left(t\right)\right)\;,\; & \left(\mathrm{dynamics}\right)\\ {\dot{\lambda }}^{*}\left(t\right) & =-\partial_{x}H\left(x^{*}\left(t\right),\lambda^{*}\left(t\right),u^{*}\left(t\right)\right)\;,\; & \left(\cos\mathrm{tate}\right)\\ H(x^{*}\left(t\right),\lambda^{*}\left(t\right),u^{*}\left(t\right)) & \geq H(x^{*}\left(t\right),\lambda^{*}\left(t\right),u)\;\forall u\in U\;.\; & \left(\mathrm{control}\right) \end{array}$$

Here, $x^{*}\left(t\right)$ and $\lambda^{*}\left(t\right)$ correspond to the optimal state and costate evolved under the action of the optimal control $u^{*}\left(t\right)$. The last equation expresses the *local* optimality condition for *u*; if the maximum does not lies on the boundary ∂U, it can be rewritten as $\partial_{u}H\left(x^{*}\left(t\right),\lambda^{*}\left(t\right),u^{*}\left(t\right)\right)=0$.

### 3.2. PMP for Affine Dynamics

We are now ready to find the optimal control u(t) for the electromechanical harvester introduced in Section 2. Before doing so, we move from the space of stochastic variables to the deterministic evolution of equal-time correlations. The dynamics in Equation (1) can be written in the form

(7) $$\dot{X}\left(t\right)=-A\left(u\left(t\right)\right)X\left(t\right)+B\xi \left(t\right)\;,\;\langle \xi_{\mu }\left(t\right)\xi_{\nu }\left(t^{\prime }\right)\rangle =\delta_{\mu \nu }\delta \left(t-t^{\prime }\right),$$

with $X\in R^{n}$ and $A,B\in R^{n\times n}$. We here study the case where *B* is constant and A(u) is a first-degree polynomial in *u*, namely

(8) $$A\left(u\right)=A_{0}+uA_{1}\;.$$

The evolution is linear: this means that if the initial state of the Fokker–Planck equation associated with Equation (7) is a Gaussian distribution centered on zero, for any later time this property will be preserved. As a consequence, the state of the system is completely determined by the covariance matrix $\Sigma_{\mu \nu }\left(t\right)=\langle X_{\mu }\left(t\right)X_{\nu }\left(t\right)\rangle$, whose evolution reads [50]

(9) $$\dot{\Sigma }\left(t\right)=-\left[A\left(u\left(t\right)\right)\Sigma \left(t\right)+\Sigma \left(t\right)A^{T}\left(u\left(t\right)\right)\right]+2D\;,\;2D=BB^{T}\;.$$

From now on, we will focus on the covariance vector $\sigma \in R^{d}$, consisting of the d=n(n+1)/2 independent components of the matrix Σ. Equation (9) leads to

(10) $$\dot{\sigma }\left(t\right)=-M\left(u\left(t\right)\right)\sigma \left(t\right)+b\;,$$

where the matrix M(u) and the vector *b* are univocally determined by *A* and *D*, respectively. Equation (10) defines the dynamics of our system, determined by its state σ. This means that the optimal control problem for the set of stochastic differential Equations (7) has been mapped to the control problem for the set of deterministic variables contained in the vector σ. The extracted power defined in Equation (2) represents the reward of our problem. Since it depends linearly on the equal-time correlations, it can be written as

(11) $$J\left[u\right]=\int_{t_{0}}^{t_{f}}dt\;u\left(t\right)\;\kappa \;\cdot \;\sigma \left(t\right)\;,$$

with κ being a constant vector. The Hamiltonian of the optimal problem then reads

(12) $$H\left(x,\lambda ,u\right)=\lambda \;\cdot \;\left[-M\left(u\right)\sigma +b\right]+u\;\kappa \;\cdot \;\sigma \;,\;M\left(u\right)=M_{0}+uM_{1}\;,$$

with $M_{0}$ and $M_{1}$ being constant matrices, because of (8). The optimal problem is then determined by $M_{0}$, $M_{1}$, *b* and κ. Explicit expressions of these objects for the harvester model (1) are provided in Appendix A; for the moment, we keep the discussion at a higher level of generality. The PMP equations read (omitting the asterisks and time dependence from now on)

(13a) $$\begin{array}{cc} \dot{\sigma } & =\partial_{\lambda }H=-M\left(u\right)\sigma +b\;, \end{array}$$

(13b) $$\begin{array}{cc} \dot{\lambda } & =-\partial_{\sigma }H=M^{T}\left(u\right)\lambda -u\kappa \;, \end{array}$$

(13c) $$\begin{array}{cc} 0 & =\partial_{u}H=-\lambda^{T}M_{1}\sigma +\kappa \;\cdot \;\sigma \;, \end{array}$$

(13d) $$\begin{array}{cc} \sigma (t_{f}) & =\sigma \left(t_{0}\right)=\sigma_{s}\;. \end{array}$$

**Figure 1:**
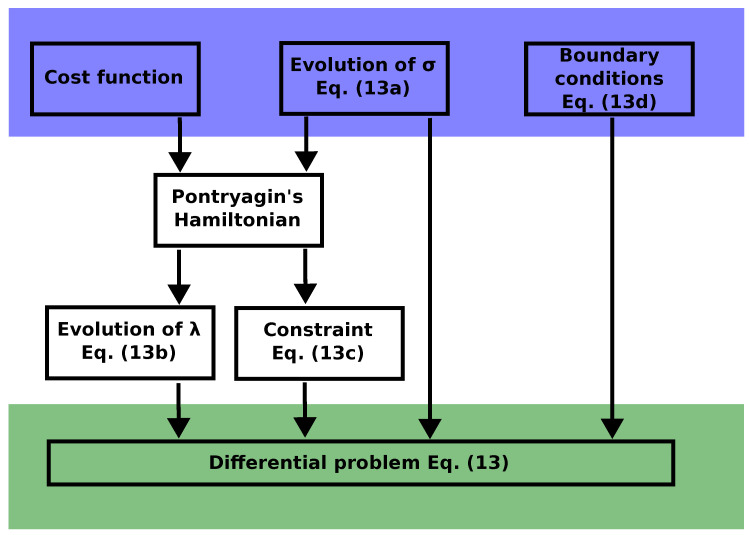
Scheme of the optimal control strategy leading to the differential system Equation (13).

Here, we included the boundary conditions (13d) for the covariance σ, defined by the same value $\sigma_{s}$. This value represents a steady state of the dynamics for $t<t_{0}$ and $t>t_{f}$, corresponding to the control value $u=u_{s}$. The control u(t) is changed in the time interval $\left(t_{0},t_{f}\right)$ and, with these prescriptions, we are guaranteed to start and to end in a stationary state purely determined by $u_{s}$, without any need for thermal relaxation. This is the main advantage of working with correlation functions, thanks to the linearity of the dynamics—i.e., the Gaussianity of the state. The control is kept at $u\equiv u_{s}$ outside the considered time interval. The reasoning we followed to obtain the differential system above is summarized in Figure 1.

Equation (13) constitutes a system of 2d ordinary differential equations for the 2d+1 variables (σ,λ,u), with 2d boundary conditions for σ (13d) and one constraint (13c) given by the stationarity along *u*. A typical solution strategy is to use the constraint (13c) to remove *u* from the dynamics and obtain a closed system of 2d equations for (σ,λ) from Equations (13a) and (13b). Unfortunately, the constraint does not depend on *u*, so it cannot be used directly to this end. But since it must vanish for all $t\in (t_{0},t_{f})$, its time derivatives will also vanish and one has

(14) $$0=\frac{d}{dt}\partial_{u}H=\lambda^{T}\left[M_{1},M_{0}\right]\sigma -\kappa^{T}M_{0}\sigma -\left(\lambda^{T}M_{1}-\kappa^{T}\right)b\;,$$

and

(15) $$\begin{array}{cc} 0 & =\frac{d^{2}}{dt^{2}}\partial_{u}H\\ \; & =\lambda^{T}\left[M,\left[M_{1},M_{0}\right]\right]\sigma +\lambda^{T}\left(\left[M_{1},M_{0}\right]-MM_{1}\right)b+\\ \; & \;-\kappa^{T}\left(u\left[M_{1},M_{0}\right]-M_{0}M\right)\sigma -\kappa^{T}\left(M_{0}-uM_{1}\right)b\\ \; & =\lambda^{T}\left[M_{0},\left[M_{1},M_{0}\right]\right]\sigma -\lambda^{T}\left(2M_{0}M_{1}-M_{1}M_{0}\right)b+\kappa^{T}M_{0}^{2}\sigma -\kappa^{T}M_{0}b+\\ \; & \;+u\left[\lambda^{T}\left[M_{1},\left[M_{1},M_{0}\right]\right]\sigma -\lambda^{T}M_{1}^{2}b+\kappa^{T}\left(2M_{0}M_{1}-M_{1}M_{0}\right)\sigma +\kappa^{T}M_{1}b\right]\;. \end{array}$$

Equation (15) gives us a *pointwise* relation for the optimal control u(t)=u(σ(t),λ(t)). The optimal solution *u* is clearly nonlinear in (σ,λ), meaning that Equations (13a) and (13b) will also become nonlinear as soon as we plug the expression for the control into them: this is the price we pay for eliminating *u* from the equations. We will see in Section 3.4 a perturbative approach to tackle this problem.

### 3.3. Is the Stationary Optimum a Global Optimum?

We know from Ref. [33] that, if one only considers a stationary control $u\left(t\right)\equiv u_{s}$, there exists a stationary optimum $u^{*}$ maximizing the stationary harvested power; the latter reads $j\left(u_{s}\right)=L\left(\sigma_{s},u_{s}\right)/\left(t_{f}-t_{0}\right)=u_{s}\;\kappa \;\cdot \;\sigma_{s}$, with $\sigma_{s}=M^{-1}\left(u_{s}\right)b$ in our notation. The optimal $u^{*}$ then satisfies

(16) $${\left.\frac{dj}{du_{s}}\right|}_{u_{s}=u^{*}}=0\;\Leftrightarrow \;{\left.\kappa^{T}\left(I-u_{s}M^{-1}\left(u_{s}\right)M_{1}\right)\sigma_{s}\right|}_{u_{s}=u^{*}}=0\;.$$

This relation determines a constant optimal $u^{*}$, and we now wonder if this optimum can also be an optimum of the dynamic control problem when u(t) is allowed to change in time. We then look at the stationary dynamics with boundary conditions $\sigma \left(t_{0}\right)=\sigma \left(t_{f}\right)=\sigma_{s}{|}_{u_{s}=u^{*}}$. Substituting $u\left(t\right)\equiv u^{*}$ into PMP Equation (13), one has

(17) $$\sigma \left(t\right)\equiv \sigma^{*}=M^{-1}\left(u^{*}\right)b\;,\;\lambda^{T}\left(t\right)\equiv {\lambda^{*}}^{T}=u^{*}\kappa^{T}M^{-1}\left(u^{*}\right)\;,$$

and the constraint then reads

(18) $$0=\partial_{u}H=-{\lambda^{*}}^{T}M_{1}\sigma^{*}+\kappa \;\cdot \;\sigma^{*}=\kappa^{T}\left(I-u^{*}M^{-1}\left(u^{*}\right)M_{1}\right)\sigma^{*}\;.$$

Since Equation (18) is the same condition satisfied by $u^{*}$ in Equation (16), the stationary optimum fulfills Pontryagin’s conditions and is a candidate to be an optimal control for the dynamic problem. However, due to the non-uniqueness of the solutions, it may happen that other protocols perform even better. We will assess the optimality of the stationary protocol under specific conditions in Section 4.

### 3.4. A Perturbative Approach to the Solution

As anticipated in Section 3.3, the standard procedure for finding optimal protocols involves expressing *u* as a function of σ and λ and subsequently solving the resulting nonlinear boundary value problem. Note that, when eliminating *u* from the system by means of Equation (15), we are not really enforcing the constraint (13c): the latter is fulfilled if Equation (15) holds $\forall t\in (t_{0},t_{f})$ *and* Equations (13c) and (14) are verified for at least one value of *t* in the same time interval. This means that we have two more constraints (local in time), and the system (13) is overdetermined. This situation is quite well known in the context of optimal control for underdamped systems [10,11,13], and can be solved formally by admitting impulsive variations in the control at the beginning and at the end of the protocol, in order to abruptly change the boundary conditions in such a way that they fulfill the constraints discussed above. While these dynamical discontinuities are physically unrealistic, several approaches to regularize them have been proposed [11,12,13,22,27,51]. These regularizations allow explicit protocols to be written down that are arbitrarily close to the optimal one and can be actually perfomed in experiments [13]. However, since the focus of this paper is mainly conceptual, we have chosen not to pursue these alternatives for the sake of simplicity, leaving them for further studies. We focus therefore on controls of the form

(19) $$u\left(t\right)=u_{0}\delta \left(t-t_{0}\right)+u_{b}\left(t\right)+u_{f}\delta \left(t-t_{f}\right)\;,$$

with $u_{b}\left(t\right)$ being the control in the bulk, $t\in (t_{0},t_{f})$, without the end times; $u_{0}=-\log\Delta_{0}$ and $u_{f}=\log\Delta_{f}$ are instead the amplitudes of the control pulses at $t=t_{0},t_{f}$, respectively, determining the discontinuities in σ and λ.

The task of solving the nonlinear problem (13) may be very challenging in most situations. A great simplification occurs if one considers the optimization problem for periodic conditions $\sigma \left(t_{0}\right)=\sigma \left(t_{f}\right)=\sigma_{s}=M^{-1}\left(u_{s}\right)b$ close to the optimal stationary solution $\sigma^{*}=M^{-1}\left(u^{*}\right)b$ discussed in Section 3.3. By eliminating u(t) from Equations (13a) and (13b) using Equation (15), and assuming that the optimal protocol remains close to $u^{*}$ at any time, we can expand the unknowns around the point $\sigma^{*}=M^{-1}\left(u^{*}\right)b$, $\lambda^{*}=u^{*}{M^{T}}^{-1}\left(u^{*}\right)\kappa$ as

(20) $$\begin{array}{cc} \sigma (t) & =\sigma^{*}+\delta \sigma \left(t\right)\;,\\ \lambda (t) & =\lambda^{*}+\delta \lambda \left(t\right)\;. \end{array}$$

The linearized system is given by

(21) $$\frac{d}{dt}\left(\begin{array}{c} \delta \sigma (t)\\ \delta \lambda (t) \end{array}\right)=-W\left(\begin{array}{c} \delta \sigma (t)\\ \delta \lambda (t) \end{array}\right)\;,$$

with $W\in R^{2n\times 2n}$ being determined by Equations (13) and (15). The solution of Equation (21) for a generic initial condition $\left(\delta \sigma \left(t_{0}^{+}\right),\delta \lambda \left(t_{0}^{+}\right)\right)$ reads

(22) $$\begin{array}{cc} \delta \sigma (t) & =U_{\sigma \sigma }\left(t,t_{0}\right)\delta \sigma \left(t_{0}^{+}\right)+U_{\sigma \lambda }\left(t,t_{0}\right)\delta \lambda \left(t_{0}^{+}\right)\;,\\ \delta \lambda (t) & =U_{\lambda \sigma }\left(t,t_{0}\right)\delta \sigma \left(t_{0}^{+}\right)+U_{\lambda \lambda }\left(t,t_{0}\right)\delta \lambda \left(t_{0}^{+}\right)\;. \end{array}$$

In the expression above, we have introduced the propagator of the dynamics

(23) $$U\left(t,t^{\prime }\right)=\exp-W(t-t^{\prime })\;,$$

and its block representation

(24) $$\;U=(\begin{array}{cc} U_{\sigma \sigma } & U_{\sigma \lambda }\\ U_{\lambda \sigma } & U_{\lambda \lambda } \end{array})\;.$$

The boundary conditions of the linearized dynamics are related to the imposed p.b.c. $\sigma_{s}$ via (see Appendix B for the derivation)

(25) $$\delta \sigma \left(t_{0}^{+}\right)=e^{-M_{1}u_{0}}\sigma_{s}-\sigma^{*}\;,\;\delta \sigma \left(t_{f}^{-}\right)=e^{M_{1}u_{f}}\sigma_{s}-\sigma^{*}\;.$$

Now, Equation (22) together with Equation (25) define a *linear* boundary value problem, which can be formally solved. As a result, we will obtain an initial value problem that satisfies the original boundary value problem without relying on numerical schemes such as the shooting method.

Using Equation (22) together with Equation (25), we can determine $\delta \lambda (t_{0}^{+})$ and $\delta \lambda (t_{f}^{-})$ as a function of $u_{0}$ and $u_{f}$. The remaining step for solving the optimal control problem consists of enforcing the validity of the constraints $\partial_{u}H=\frac{d}{dt}\partial_{u}H=0$ at $t=t_{0}^{+}$ and $t=t_{f}^{-}$. In the perturbative approach we consider here, these constraints can be reduced to a system of coupled algebraic equations. Indeed, by virtue of the Cayley–Hamilton theorem [52,53], every function f(·) of a d×d matrix *G* can be expressed as a polynomial of degree d−1 in *G*, that is

(26) $$f\left(G\right)=\sum_{l=0}^{d-1}g_{l}G^{l}\;.$$

Thus, by inserting f(·)=exp(·) and $G=u_{0}M_{1}$ or $G=u_{f}M_{1}$ in Equation (25), we obtain

(27) $$\begin{array}{cc} \; & \delta \sigma \left(t_{0}^{+}\right)=\sum_{l=0}^{d-1}{\left(-1\right)}^{l}m_{l}M_{1}^{l}\sigma_{s}u_{0}^{l}-\sigma^{*}\;, \end{array}$$

(28) $$\begin{array}{cc} \; & \delta \sigma \left(t_{f}^{-}\right)=\sum_{l=0}^{d-1}m_{l}M_{1}^{l}\sigma_{s}u_{f}^{l}-\sigma^{*}\;, \end{array}$$

with $m_{l}$ being the expansion coefficients of the exponential function. Solving Equation (22) to express $\delta \lambda (t_{0}^{+})$ and $\delta \lambda (t_{f}^{-})$ as functions of $\delta \sigma (t_{0}^{+})$, $\delta \sigma (t_{f}^{-})$, and substituting Equations (27) and (28) for the latter, we obtain

(29) $$\begin{array}{cc} \; & \lambda \left(t_{0}^{+}\right)=\sum_{l=0}^{d-1}\left[\lambda_{l}^{\left(+,0\right)}u_{0}^{l}+\lambda_{l}^{\left(+,f\right)}u_{f}^{l}\right]\;, \end{array}$$

(30) $$\begin{array}{cc} \; & \lambda \left(t_{f}^{-}\right)=\sum_{l=0}^{d-1}\left[\lambda_{l}^{\left(-,0\right)}u_{0}^{l}+\lambda_{l}^{\left(-,f\right)}u_{f}^{l}\right]\;, \end{array}$$

where $\lambda_{l}^{\left(\pm ,0\right)}$ and $\lambda_{l}^{\left(\pm ,f\right)}$ are vectorial coefficients. Since $\delta \sigma (t_{0}^{+})$, $\delta \sigma (t_{f}^{-})$, $\delta \lambda (t_{0}^{+})$ and $\delta \lambda (t_{f}^{-})$ are all polynomials of degree d−1 *at most* in $u_{0}$ and $u_{f}$, and the constraints $\partial_{u}H=\frac{d}{dt}\partial_{u}H=0$ involve only quadratic nonlinearities, finding the optimal protocol reduces to solving two coupled algebraic equations of degree 2(d−1) at most, i.e.,

(31) $$\sum_{l=0}^{2(d-1)}c_{l}^{\left(1\right)}u_{0}^{l}u_{f}^{2(d-1)-l}=0\;,\;\sum_{l=0}^{2(d-1)}c_{l}^{\left(2\right)}u_{0}^{l}u_{f}^{2(d-1)-l}=0\;,$$

with $c_{l}^{\left(1\right)}$ and $c_{l}^{\left(2\right)}$ being coefficients determined by the problem (i.e., by the matrix *M*). In general, the above system admits multiple solutions, and the optimal control must be determined as the one that maximizes the reward

(32) $$J\left[u\right]=\int_{0}^{t_{f}}dt\;u\left(t\right)\kappa \;\cdot \;\sigma \left(t\right)=\kappa \;\cdot \;\left[\left(e^{M_{1}u_{f}}-e^{-M_{1}u_{0}}\right)M_{1}^{-1}\sigma_{s}+\int_{0}^{t_{f}}dt\;u_{b}\left(t\right)\sigma \left(t\right)\right]\;,$$

where the first terms stem from discontinuities while the last from the bulk dynamics (see Appendix B). In the following, we will apply the aforementioned procedure to the energy harvester model (1) introduced in Section 2.

## 4. Results

The framework introduced in Section 3 to identify optimal protocols for affine dynamics can be applied to the energy harvester model (1). A standard analysis of the physical dimensions involved (see Appendix A) allows us to rewrite the evolution in dimensionless units as

(33) $$\begin{array}{cc} \dot{x} & =v\\ \dot{v} & =-\alpha x-\beta v-I+\xi \\ \dot{I} & =v-\varepsilon I\\ \langle \xi \left(t\right)\xi \left(t^{\prime }\right)\rangle & =2\delta (t-t^{\prime })\;, \end{array}$$

with the control *u* acting on

ε=ζ+u.

Here, α, β and ζ are constant parameters, while *u* is a time-dependent control taking positive values. We assume that *u* can be changed with arbitrary speed and precision: this is of course an idealization, since our ability to control *u* actually depends on the operating range of the potentiometer in use. In Appendix A, we also show that the stationary extracted power in dimensionless units reads

(34) $$P_{s}\left(u_{s}\right)=\frac{u_{s}}{\varepsilon_{s}+\beta \left(1+\alpha +\varepsilon_{s}\left(\beta +\varepsilon_{s}\right)\right)}$$

(with $\varepsilon_{s}=\zeta +u_{s}$), and is maximized by

(35) $$u^{*}=\sqrt{\frac{\alpha \beta +(\beta +\zeta )(1+\beta \zeta )}{\beta }}\;.$$

As discussed in Section 3.3, the stationary protocol $u=u^{*}$ is a solution of (dynamical) Pontryagin’s optimal problem (13), with boundary conditions $\sigma \left(0\right)=\sigma^{*}$ and $\sigma \left(t_{f}\right)=\sigma^{*}$. In other words, if the system is initially prepared in the stationary state $\sigma^{*}$ and needs to be brought back to $\sigma^{*}$ in time $t_{f}$, the intuitive strategy of keeping the potentiometer fixed at $u=u^{*}$ does fulfill Pontryagin’s necessary condition (for any time interval $t_{f}$). However, the solution of problem (13) is not unique in general, so there is no guarantee that such prescription is actually the best. As a matter of fact, in the considered case better solutions can be found, at least for some choices of the parameters: we will come back to this point in the following “An Explicit Example: The Case α=0” Section.

All considerations made in Section 3 can be applied, in principle, to model (33). The explicit calculations may, however, turn out to be quite involved, due to the high dimensionality of system (13): here, σ and λ are 6-dimensional vectors, and hence *W* and *U* turn out to be 12×12 matrices. Our goal here is not to find the solution of the optimal problem for a realistic choice of the parameters, but rather to provide a proof of principle of the usefulness of the method and the possibility of having dynamical protocols that are more efficient than the statical one $u=u^{*}$. Therefore, in the following we focus on a particular regime for which we are able to provide explicit solutions. A more systematic exploration of the several regimes of the model is left as the subject for future research work.

### An Explicit Example: The Case α=0

A considerable simplification in model (33) arises when considering the particular case α=0. From a physical point of view, this choice corresponds to an overdamped limit, in which the relaxation dynamics due to the presence of viscous friction are much faster than the oscillatory dynamics of the spring (i.e., $\tau_{\theta }\ll \tau_{k}$: see Appendix A for details). In this limit, the dynamics of *v* and *I* are completely decoupled from those of *x*. Moreover, the reward function (2) does not depend on *x* in any way. We can therefore focus on the variables *v* and *I* only, and uniquely characterize the state of the system by the covariances $\langle v^{2}\rangle$, $\langle vI\rangle$ and $\langle I^{2}\rangle$. The dimension of σ reduces to 3, as well as that of λ. Equations (5) and (10) can be written in this smaller space by taking (see Equation (A4) in Appendix A)

(36) $$M_{0}=\left(\begin{array}{ccc} 2\beta & 2 & 0\\ -1 & \beta +\zeta & 1\\ 0 & -2 & 2\zeta \end{array}\right)\;,\;M_{1}=\left(\begin{array}{ccc} 0 & 0 & 0\\ 0 & 1 & 0\\ 0 & 0 & 2 \end{array}\right)\;,\;b=\left(\begin{array}{c} 2\\ 0\\ 0 \end{array}\right)\;,\;\kappa =\left(\begin{array}{c} 0\\ 0\\ 1 \end{array}\right)\;.$$

We limit our attention to the case of boundary conditions corresponding to equal stationary states,

(37) $$\sigma \left(0\right)=\sigma \left(t_{f}\right)=\sigma_{s}\left(u_{s}\right)\;.$$

This case is particularly relevant, because it allows us to repeat the process cyclically. To exploit the perturbative approach outlined in Section 3.4, we also need to choose $u_{s}$ close enough to $u^{*}$, and carefully verify a posteriori that the protocol u(t) also connects the initial and final values of σ in the original, true dynamics.

**Figure 2:**
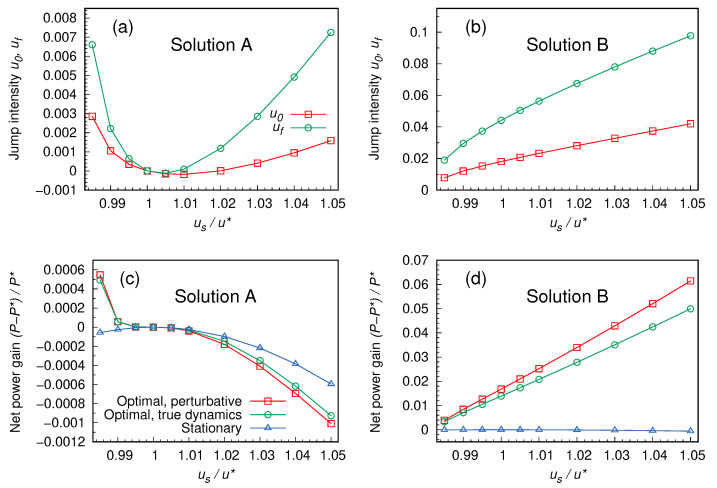
Characterization of the solutions of PMP. Panels (**a**,**b**) show the intensity of the infinite discontinuities $u_{0}$ and $u_{f}$ occurring at the beginning and at the end of the protocol in the two physically admissible solutions A and B of system (31). Different choices of the stationary control $u_{s}$, fixing the boundary conditions (37), are considered. Panels (**c**,**d**) account for the corresponding net power gain (or loss) with respect to the stationary strategy $u=u^{*}$. The red squares refer to the value of the average power computed within the perturbative approach. Green circles are obtained by plugging the solution protocol *u* into the original (non-perturbative) dynamics, and computing the average power of the process (see the caption of Figure 4 for details). Of course, in this case the final state $\sigma (t_{f})$ will not match the prescribed boundary condition exactly—see Figure 4. The distance between the two curves is an indicator of the quality of the perturbative approximation. Finally, the blue triangles represent, for reference, the power obtained with a stationary protocol $u=u_{s}$. Parameters: α=0, β=1, ζ=2 and $t_{f}=0.25$.

When (numerically) solving system (31), one obtains up to 12 different solutions for the unknowns $\left(u_{0},u_{f}\right)$. However, most of them are not physically admissible, since they imply complex or negative values of *u* in the time interval $\left[0,t_{f}\right]$. In particular, for the range of parameters explored in Figure 2, we only find two admissible solutions: we call “A” the one closer to $\left(u_{0}=0,u_{f}=0\right)$ (Figure 2a), and “B” the one farther from it (Figure 2b). We actually also classify as “A” some solutions, found in the range $1<u_{s}/u^{*}\leq 1.02$, which involve negative values of $u_{0}$ and/or $u_{f}$, and are therefore not physical. Notice that the optimal stationary solution $u=u^{*}$ is solution A for the case with boundary conditions $\sigma \left(0\right)=\sigma \left(t_{f}\right)=\sigma^{*}$.

We therefore find that, as shown in Figure 2c,d, it is possible to find dynamical protocols that perform better than the optimal stationary one. In particular, solution A with $u_{s}<u^{*}$ is able to extract more power than the optimal stationary one, $P^{*}=P_{s}\left(u^{*}\right)$. Solution B, in the explored parameter range, is even better performing. We verified that a non-negligible contribution to the extracted power comes from the impulsive changes in *u* happening at the beginning and at the end of the protocol. Without them, the solutions would not outperform the stationary strategy. Even if we do not have a definitive argument for this, we conjecture that the presence of steep changes in the control protocol, giving access to a broad space of possible solutions, is crucial in this sense.

**Figure 3:**
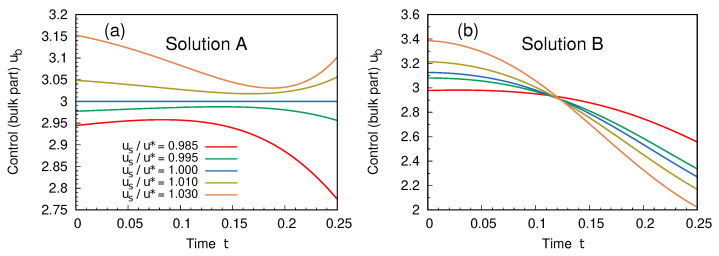
Bulk part $u_{b}$ of the protocol, for the two solutions A (**a**) and B (**b**), as a function of time. Different boundary conditions are considered. Parameters as in Figure 2.

**Figure 4:**
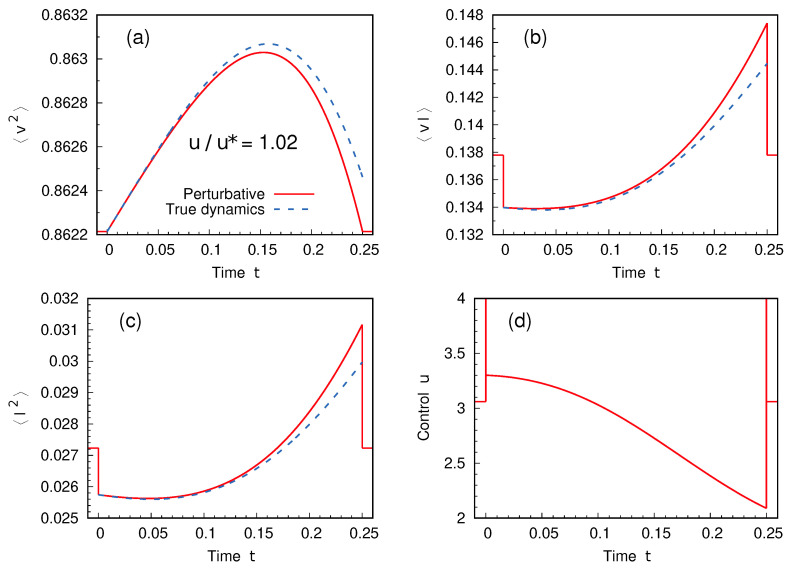
Dynamics of the system within solution B for boundary conditions fixed by $u_{s}=1.02\;u^{*}$. Panels (**a**–**c**) show the evolution of the elements of the vector σ (the covariances $\langle v^{2}\rangle$, $\langle vI\rangle$ and $\langle I^{2}\rangle$), as computed in the perturbative approach (red solid curves). By inserting the solution protocol *u*, shown in panel (**d**), back into the original dynamics (33), it is possible to compute the true behavior of the system under the prescribed protocol (computation has been carried out using an explicit Runge–Kutta integration scheme of 4-th order): this evolution is represented by the blue dashed curves in panels (**a**–**c**). While it is expected that the two sets of curves do not overlap, the fact that they stay close is a consistency check on our perturbative approximation. Parameters as in Figure 2.

The bulk part of the protocol, $u_{b}$, is shown in the two cases in Figure 3. As expected, solution A reduces to the stationary one for $u_{s}=u^{*}$. The detailed dynamics of solution B for one choice of the boundary conditions is finally shown in Figure 4, where the quality of the perturbative approach is also examined.

## 5. Discussion

In this work, we have presented a proof of concept that dynamical control methods can be used to improve the efficiency of an energy harvester model driven by broadband vibrations. We have considered a linear model, which has been shown to well reproduce the dynamics of electromagnetic and piezoelectric energy harvesters, for which both theoretical and experimental studies are available [30,33]. This kind of system is described by a Langevin equation for the movable part, coupled with a deterministic equation for the current flowing in the output circuit. We have shown that changing in time the value of the load resistance can lead to larger work extraction. In particular, we were able to find optimal dynamical solutions by mean of Pontryagin’s Principle. In general, the prescribed condition leads to a system of ordinary differential equations, which is quite challenging to solve. However, it is always possible to linearize the equations around the optimal stationary solution and search for protocols that do not differ too much from it.

The kind of optimal protocols found in this work may appear rather difficult to implement at a practical level, since they involve abrupt discontinuities; we leave for future studies the regularization of these solutions. Such regularization is expected to lead to realistic, implementable protocols, at the price of lower harvesting power. Since the discontinuities only appear at the beginning and at the end of the protocols, an interesting future perspective would be considering the optimization problem in the limit $t_{f}\to \infty$ and checking whether it is possible to find a periodic optimal protocol whose period is related to some characteristic time of the model. Another important point that should be addressed when considering realistic implementations of our protocols is the energetic cost of varying the circuit resistance. This is related to the maximum power point tracking (MPPT) problem [54]. At the theoretical level, it would be interesting to introduce an objective function that also includes the energy cost of the parameter tuning in our system.

Finally, we point out that a promising alternative approach that can be pursued on the basis of the present work is to consider optimal updates of the control variable as a function of real-time measurements performed on the system (i.e., closed feedback loop) within the framework of dynamic programming [55].

## Data Availability

Numerical data presented in Section 4 are available from the authors upon reasonable request.

## Appendix A. Energy Harvester Model in Dimensionless Units

We discuss here how to rewrite the dynamics (1) into a more compact form, passing to dimensionless variables. We notice that there are four dimensional units to be fixed:

1. Mass M=1;
2. Time $\tau =\tau_{\theta }=\sqrt{ML_{c}}/\theta =1$;
3. Length $\ell =\sqrt{D_{0}\tau^{3}}=1$;
4. Current intensity $\iota =\theta \ell /L_{C}=1$.

The dimensionless dynamic variables are then $x^{*}=x/\ell$, $t^{*}=t/\tau$ and $I^{*}=I/\iota$. We will drop the asterisks from now on. In these new variables, the dynamics read

(A1) $$\begin{array}{cc} \dot{x} & =v\\ \dot{v} & =-\alpha x-\beta v-I+\xi \\ \dot{I} & =v-\varepsilon I\\ \langle \xi \left(t\right)\xi \left(t^{\prime }\right)\rangle & =2\delta (t-t^{\prime })\;. \end{array}$$

The dimensionless coefficients read

(A2) $$\alpha =\frac{k_{s}\tau_{\theta }^{2}}{M}={\left(\frac{\tau_{\theta }}{\tau_{k}}\right)}^{2}\;,\;\beta =\frac{\gamma \tau_{\theta }}{M}=\frac{\tau_{\theta }}{\tau_{v}}\;,\;\varepsilon =\frac{\left(R_{C}+R\right)\tau_{\theta }}{L_{C}}=\frac{\tau_{\theta }}{\tau_{C}}+\frac{\tau_{\theta }}{\tau_{R}}=\zeta +u\;,$$

having defined the characteristic times $\tau_{k}=\sqrt{M/k_{s}}$ (elastic response), $\tau_{v}=M/\gamma$ (viscous friction), $\tau_{\theta }$ (electromechanical coupling), $\tau_{C}=L_{C}/R_{C}$ (coil) and $\tau_{R}=L_{C}/R$ (resistance).

Just to give an idea of the order of magnitude of these coefficients, we report the experimental values used in [33] in Table A1 and the characteristic times and dimensionless parameters in Table A2. We stress that the aim of this paper is not to provide solutions for a realistic system; therefore, in our explicit examples we will use instead a more idealized, easier to treat set of parameters.

**Table A1:** Experimental values of the electromechanical harvester, measured in [33].

*M* (kg)	γ (kg/s)	$k_{s}$ (N/m)	θ (N/A)	$L_{C}$ (H)	$R_{C}$ (Ω)
0.048(5)	1.80(5)	18,810(50)	29.9(5)	0.124(5)	227.6(5)

**Table A2:** Characteristic times (in seconds) and dimensionless parameters, neglecting errors.

$\tau_{v}$	$\tau_{k}$	$\tau_{\theta }$	$\tau_{C}$	α	β	ζ
2.67 × 10^−2^	1.60 × 10^−3^	2.58 × 10^−3^	5.44 × 10^−4^	3.22	9.66 × 10^−2^	4.74

Using the Ornstein–Uhlenbeck formalism adopted in [33], one obtains the matrices

(A3) $$\dot{X}=-AX+\eta \;,\;A=\left(\begin{array}{ccc} 0 & -1 & 0\\ \alpha & \beta & 1\\ 0 & -1 & \varepsilon \end{array}\right)\;,\;D=\left(\begin{array}{ccc} 0 & 0 & 0\\ 0 & 1 & 0\\ 0 & 0 & 0 \end{array}\right)\;.$$

We stress that in the formalism of Equations (10) and (12), this means that the evolution of the state vector

$$\sigma ={\left(\begin{array}{cccccc} \sigma_{XX} & \sigma_{XV} & \sigma_{XI} & \sigma_{VV} & \sigma_{VI} & \sigma_{II} \end{array}\right)}^{T}$$

is determined by

(A4) $$M_{0}=\left(\begin{array}{cccccc} 0 & -2 & 0 & 0 & 0 & 0\\ \alpha & \beta & 1 & -1 & 0 & 0\\ 0 & -1 & \zeta & 0 & -1 & 0\\ 0 & 2\alpha & 0 & 2\beta & 2 & 0\\ 0 & 0 & \alpha & -1 & \beta +\zeta & 1\\ 0 & 0 & 0 & 0 & -2 & 2\zeta \end{array}\right)\;,\;M_{1}=\left(\begin{array}{cccccc} 0 & 0 & 0 & 0 & 0 & 0\\ 0 & 0 & 0 & 0 & 0 & 0\\ 0 & 0 & 1 & 0 & 0 & 0\\ 0 & 0 & 0 & 0 & 0 & 0\\ 0 & 0 & 0 & 0 & 1 & 0\\ 0 & 0 & 0 & 0 & 0 & 2 \end{array}\right)\;,\;b=\left(\begin{array}{c} 0\\ 0\\ 0\\ 2\\ 0\\ 0 \end{array}\right)\;,\;\kappa =\left(\begin{array}{c} 0\\ 0\\ 0\\ 0\\ 0\\ 1 \end{array}\right)\;.$$

In the case of constant control $u\left(t\right)=u_{s}$ (and, as a consequence, $\varepsilon \left(t\right)=\varepsilon_{s}$), the stationary covariance matrix $\Sigma_{s}$ of entries $\sigma_{\mu \nu }^{s}=\langle X_{\mu }X_{\nu }\rangle$, with μ,ν∈{X,V,I}, satisfies the condition

$$2D=A\Sigma_{s}+\Sigma_{s}A^{T}$$

and the components read

(A5) $$\begin{array}{cccc} \sigma_{XX}^{s} & =\frac{\alpha +\varepsilon (\beta +\varepsilon )}{\alpha [\varepsilon +\beta (1+\alpha +\varepsilon (\beta +\varepsilon ))]}\;, & \sigma_{XV}^{s} & =0\;,\\ \sigma_{VV}^{s} & =\frac{1+\alpha +\varepsilon (\beta +\varepsilon )}{\varepsilon +\beta (1+\alpha +\varepsilon (\beta +\varepsilon ))}\;, & \sigma_{XI}^{s} & =\frac{1}{\varepsilon +\beta (1+\alpha +\varepsilon (\beta +\varepsilon ))}\;,\\ \sigma_{II}^{s} & =\frac{1}{\varepsilon +\beta (1+\alpha +\varepsilon (\beta +\varepsilon ))}\;, & \sigma_{VI}^{s} & =\frac{\varepsilon }{\varepsilon +\beta (1+\alpha +\varepsilon (\beta +\varepsilon ))}\;. \end{array}$$

The stationary extracted power (in dimensionless units) reads

(A6) $$P_{s}=\frac{\tau_{\theta }^{3}}{M\ell^{2}}R\langle I^{2}\rangle =\frac{\tau_{\theta }}{\tau_{R}}\langle I^{2}\rangle =u\sigma_{II}^{s}=\frac{u}{\varepsilon +\beta (1+\alpha +\varepsilon (\beta +\varepsilon ))}$$

and is maximized by the choice (recall that ε=ζ+u)

(A7) $$u^{*}=\sqrt{\frac{\alpha \beta +(\beta +\zeta )(1+\beta \zeta )}{\beta }}\;.$$

## Appendix B. Discontinuous Protocol: Boundary Conditions and Power Extraction

Infinite discontinuities in control are convenient for analytical purposes but must be properly interpreted. Let us consider a control u(t) of the form

(A8) $$u\left(t\right)=u_{0}\delta_{\epsilon }\left(t-t_{0}\right)+u_{b}\left(t\right)+u_{f}\delta_{\epsilon }\left(t-t_{f}\right)$$

where $0<\epsilon \ll t_{f}-t_{0}$. Here, we assume that $u_{b}\left(t\right)\neq 0$ if and only if $t\in [t_{0}+\epsilon ,t_{f}-\epsilon ]$. The function $\delta_{\epsilon }\left(t\right)$ denotes a generic distribution fulfilling the property

(A9) $$\int_{0}^{\epsilon }dt\;\delta_{\epsilon }\left(t\right)=1,$$

which converges to the Dirac delta function when ϵ→0. As a consequence, in Equation (13a) the terms proportional to u(t) represent the leading contribution to $\dot{\sigma }$ for $t\in [t_{0},t_{0}+\epsilon )$ and $t\in (t_{f}-\epsilon ,t_{f}]$, that is

(A10) $$\dot{\sigma }≃-uM_{1}\sigma \Rightarrow \sigma \left(t\right)≃\left\{\begin{array}{cc} e^{-M_{1}\int_{t_{0}}^{t}u\left(t\right)}\sigma \left(t_{0}\right)\;;\; & t\in [t_{0},t_{0}+\epsilon )\\ e^{-M_{1}\int_{t_{f}-\epsilon }^{t}u\left(t\right)}\sigma \left(t_{f}-\epsilon \right)\;;\; & t\in (t_{f}-\epsilon ,t_{f}] \end{array}\right.$$

By evaluating the above expressions in $t=t_{0}+\epsilon$ or $t=t_{f}$, respectively (also using Equation (A9)), we obtain

(A11) $$\begin{array}{cc} \; & \sigma \left(t_{0}^{+}\right)=\lim_{\epsilon \to 0}\sigma \left(t_{0}+\epsilon \right)=e^{-M_{1}u_{0}}\sigma \left(t_{0}\right) \end{array}$$

(A12) $$\begin{array}{cc} \; & \sigma \left(t_{f}^{-}\right)=\lim_{\epsilon \to 0}\sigma \left(t_{f}-\epsilon \right)=e^{M_{1}u_{f}}\sigma \left(t_{f}\right)\;, \end{array}$$

which coincide with expressions (25) given in the main text. Let us now focus on the effects of discontinuities on the reward

$$J\left[u\right]=\int_{t_{0}}^{t_{f}}dt\;u\left(t\right)\kappa \;\cdot \;\sigma \left(t\right)=\kappa \;\cdot \;\left[\int_{t_{0}}^{t_{f}}dt\;u\left(t\right)\sigma \left(t\right)\right]\;.$$

Note that the integral above can be split into

(A13) $$\int_{t_{0}}^{t_{f}}dt\;u\left(t\right)\sigma \left(t\right)=\lim_{\epsilon \to 0}\left[\int_{t_{0}}^{t_{0}+\epsilon }dt\;u\left(t\right)\sigma \left(t\right)+\int_{t_{0}+\epsilon }^{t_{f}-\epsilon }dt\;u\left(t\right)\sigma \left(t\right)+\int_{t_{f}-\epsilon }^{t_{f}}dt\;u\left(t\right)\sigma \left(t\right)\right]\;.$$

Noting that

$$u\left(t\right)\sigma \left(t\right)=-M_{1}^{-1}\dot{\sigma }$$

for $t\in [t_{0},t_{0}+\epsilon )$ and $t\in (t_{f}-\epsilon ,t_{f}]$, Equation (A13) can be written as

(A14) $$\begin{array}{cc} \int_{t_{0}}^{t_{f}}dt\;u\left(t\right)\sigma \left(t\right) & =\int_{t_{0}}^{t_{f}}dt\;u_{b}\left(t\right)\sigma \left(t\right)-\lim_{\epsilon \to 0}M_{1}^{-1}\left[\int_{t_{0}}^{t_{0}+\epsilon }dt\;\dot{\sigma }\left(t\right)+\int_{t_{f}-\epsilon }^{t_{f}}dt\;\dot{\sigma }\left(t\right)\right] \end{array}$$

(A15) $$\begin{array}{cc} \; & =\int_{t_{0}}^{t_{f}}dt\;u_{b}\left(t\right)\sigma \left(t\right)-M_{1}^{-1}\left[\sigma \left(t_{0}^{+}\right)-\sigma \left(t_{0}\right)+\sigma \left(t_{f}\right)-\sigma \left(t_{f}^{-}\right)\right]\;. \end{array}$$

Combining Equation (A12) with Equation (A15) leads to

(A16) $$J\left[u\right]=\kappa \;\cdot \;\left[\int_{t_{0}}^{t_{f}}dt\;u_{b}\left(t\right)\sigma \left(t\right)+\left(I-e^{-M_{1}u_{0}}\right)M_{1}^{-1}\sigma \left(t_{0}\right)+\left(e^{M_{1}u_{f}}-I\right)M_{1}^{-1}\sigma \left(t_{f}\right)\right]\;,$$

which corresponds to Equation (32) for $\sigma \left(t_{0}\right)=\sigma \left(t_{f}\right)=\sigma_{s}$.
